# Metabolomic Analysis of Different Parts of Black Wax Gourd (*Cucurbita pepo*)

**DOI:** 10.3390/foods14061046

**Published:** 2025-03-19

**Authors:** Jun Li, Haocheng Liu, Yujuan Xu, Jiguo Yang, Yuanshan Yu, Jing Wen, Dasen Xie, Yujuan Zhong, Jijun Wu, Manqin Fu

**Affiliations:** 1 Sericultural & Argi-Food Research Institute, Guangdong Academy of Agricultural Sciences/Key Laboratory of Functional Foods, Ministry of Agriculture and Rural Affairs/Guangdong Key Laboratory of Agricultural Products Processing, No. 133 Yiheng Street, Dongguanzhuang Road, Tianhe District, Guangzhou 510610, China; junli245@126.com (J.L.); ansishc@163.com (H.L.); guoshuxuyujuan@163.com (Y.X.); yuyuanshan@gdaas.cn (Y.Y.); jingw988@163.com (J.W.); 2 School of Food Science and Engineering, South China University of Technology, Guangzhou 510641, China; yangjg@scut.edu.cn; 3 Guangdong Key Laboratory for New Technology Research of Vegetables, Vegetable Research Institute, Guangdong Academy of Agricultural Sciences, Guangzhou 510640, China; xiedasen@126.com (D.X.); zhongyujuan@gdaas.cn (Y.Z.)

**Keywords:** black wax gourd, functional activity, nutritive value, UPLC-MS/MS

## Abstract

This study employed ultra-performance liquid chromatography–tandem mass spectrometry (UPLC-MS/MS) combined with multivariate analysis to investigate tissue-specific metabolic profiles in the peel, pulp, and seeds of black wax gourd (Benincasa hispida). A total of 1020 metabolites were identified, including 520 primary metabolites (e.g., amino acids, lipids, and organic acids) and 500 secondary metabolites (e.g., phenolic acids, flavonoids, and alkaloids). Significant metabolic divergence was observed across tissues: 658, 674, and 433 differential metabolites were identified between the peel and the pulp, the peel and the seeds, and the pulp and the seeds, respectively. Unique metabolites such as methyl 5-glucosyloxy-2-hydroxybenzoate and 3,5-di-*O*-caffeoylquinic acid were exclusive to the peel, while 4-*O*-(6′-*O*-glucosyl-imino)-4-hydroxybenzyl alcohol and fertaric acid were specific to the seeds. KEGG pathway enrichment revealed distinct metabolic priorities: flavonoids and phenolic acids dominated in the peel, amino acids and phenylpropanoids in the pulp, and nucleotides and lipids in the seeds. The peel exhibited the highest secondary metabolite abundance (14.27%), whereas the seeds accumulated the most primary metabolites (26.62%), including essential amino acids like L-tryptophan and functional lipids such as linoleic acid. These findings underscore the nutritional and bioactive potential of underutilized by-products (peel and seeds), providing a biochemical foundation for valorizing wax gourd tissues in the food, pharmaceutical, and agricultural industries.

## 1. Introduction

The wax gourd (*Benincasa hispida* Cogn.) belongs to the genus Benincasa in the family Cucurbitaceae, also called white gourd and pillow gourd, and is an annual plant of the Cucurbitaceae, native to Southern China and East India [1]. It is a summer and autumn vegetable widely distributed in tropical, subtropical, and temperate regions of Asia and has become an ideal raw material for modern agricultural product processing and a main vegetable to regulate a balanced market supply because of its wide planting area, stable yield, and rich nutrients [2,3,4]. The black wax gourd is the main plant cultivar in Guangdong, Guangxi, Hainan, and other provinces in China, and it is the main vegetable transported north from the south, which is of great significance to local farmers’ income and rural economic development [5,6]. Meanwhile, it possesses the potential to promote planting in wax gourd production areas throughout the country due to its developed root system, strong root absorption, and stem branching ability [7], resulting in a gradual increase in production. However, at present, China’s wax gourds are mainly sold fresh, with low added value. In addition, 8% of peels and 4% of seeds are discarded as general waste during primary processing, resulting in resource waste and environmental pollution [8,9,10]. Therefore, it is of great significance for promoting the high-quality development of the wax gourd industry to elucidate the differences in metabolites in different parts of black wax gourd and the basis of its nutrient-active substances, as well as fully tapping the utilization value of different by-products.

The thorough processing of by-products is one of the emerging trends in the food industry and an approach to increase the added value [11,12]. The by-products in fruits and vegetables are the key to the natural sources of active ingredients, and their content is even larger than the edible part. This is the case with the by-products of wax gourds, which have high nutritional value. For instance, the wax gourd peel is rich in flavonoids, polyphenols, polysaccharides, alkaloids, and other active ingredients [5,13,14], and dried wax gourd peel is also one of the Chinese medicinal materials included in the pharmacopoeia, with diuresis and detumescence effects and use in summer heat and thirst treatments. Wax gourd seeds contain saponins, fatty acids, and tiny amounts of triterpenoids, proteins, steroids, and other active substances [15]. In addition, modern scientific studies have shown that wax gourd seed extracts can promote mucus secretion, inhibit histamine secretion, and have an anti-tumor effect by enhancing the immune response [16,17]. However, current research on wax gourds mainly focuses on the growing environment, genetic analysis of traits, and planting techniques [18]. For instance, Xue et al. identified nine quantitative trait loci (QTLs) associated with weight, length, diameter, and pulp thickness on chromosomes 3, 4, 5, 6, 9, 10, and 11 during the genetic analysis of wax gourd traits [19]. Furthermore, Su et al.’s study revealed BhSSC2.1 as a pivotal gene regulating the soluble solid content. These findings were based on the integrated analysis of metabolomics and transcriptomics conducted on two wax gourd peel varieties, namely B214 and B227 [20]. Primary metabolites are essential compounds for organisms to perform basic life activities, and these include amino acids, nucleic acids, carbohydrates, and lipids. They primarily participate in cellular energy metabolism and biosynthesis. In contrast, secondary metabolites are compounds produced during specific developmental stages or in response to environmental stimuli, and they include flavonoids, phenolic acids, and alkaloids. These compounds often exhibit specific physiological functions, such as antioxidant, anti-inflammatory, and anticancer activities. The nutritional quality of wax gourds mainly depends on their primary metabolites, such as amino acids, nucleotides, polysaccharides, lipids, and vitamins, whereas the secondary metabolites contain physiologically active substances which can have a great impact on human health [21]. In contrast, differences in metabolites between different parts of wax gourds have not been reported yet.

Therefore, to better analyze the nutritional characteristics of black-skinned wax gourds, this study employed ultra-performance liquid chromatography–tandem mass spectrometry (UPLC-MS/MS)-based metabolomics to characterize tissue-specific metabolic variations between three anatomical compartments: the peel, the pulp, and the seeds. Through the systematic identification and quantification of both primary metabolites (nutritional components) and specialized secondary metabolites (bioactive compounds), we conducted comparative metabolic profiling to elucidate compartment-specific accumulation patterns. A subsequent pathway enrichment analysis using the KEGG database revealed critical metabolic divergences. These findings establish a biochemical foundation for understanding the differential nutritional and functional properties of wax gourd tissues, while providing scientific rationale for valorizing agricultural by-products through targeted utilization strategies in the food and phytochemical industries.

## 2. Materials and Methods

### 2.1. Samples and Preparation

The wax gourd used in this study was supplied by the Vegetable Research Institute of the Guangdong Academy of Agricultural Sciences. This variety, known as Guishu No. 3, is a new cultivar with potential for widespread cultivation in winter melon production areas nationwide. Methanol and acetonitrile, both of chromatographic purity, were purchased from Thermo Fisher Scientific Inc. (Merck KGaA, Darmstadt, Germany), and the standard dimethyl sulfoxide (DMSO) (≥99%) was purchased from Sigma-Aldrich (Saint Louis, MO, USA). Unless otherwise stated, all other reagents were of analytical grade and purchased from Beijing Chemical Co. (Beijing, China). Ultrapure water was prepared using a Milli-Q system purchased from Millipore (Billerica, MA, USA).

Uniform black-skinned wax gourds were selected, with their appearance, color, and other characteristic indices detailed in Supplementary Table S1. The exterior of the wax gourds was cleaned, and the peel, pulp, and seeds were collected separately. Each of these three parts was subjected to vacuum freeze-drying and subsequently ground into powder. Precisely 0.10 g of powder from each part was weighed and dissolved in 1.20 mL of extraction solvent (70% methanol). The resulting sample solutions were vortexed for six intervals of 30 min each, followed by incubation at 4 °C overnight. The samples were then centrifuged for 10 min at 12,000 rpm. The supernatants were filtered through a 0.22 μm pore size microporous membrane, and the filtrates were stored in injection vials for subsequent UPLC-MS/MS analysis.

### 2.2. UPLC-MS/MS Acquisition Analysis

#### 2.2.1. UPLC Conditions

The sample extracts were analyzed using an UPLC-ESI-MS/MS system (UPLC, SHIMADZU Nexera X2-Shimadzu-Kyoto, Japan, https://www.shimadzu.com.cn/, accessed on 1 October 2024); MS, Applied Biosystems 4500 Q TRAP, https://www.thermofisher.cn/cn/zh/home/brands/applied-biosystems.html, accessed on 1 October 2024). The analytical conditions were as follows: UPLC employed a column, Agilent SB-C18 (1.8 µm, 2.1 mm × 100 mm). The mobile phase consisted of solvent A, pure water with 0.1% formic acid, and solvent B, acetonitrile with 0.1% formic acid. Sample measurements were performed with a gradient program that employed the starting conditions of 95% A and 5% B. Within 9 min, a linear gradient to 5% A and 95% B was programmed, and a composition of 5% A and 95% B was kept for 1 min. Subsequently, a composition of 95% A and 5.0% B was adjusted over 1.1 min and kept for 2.9 min. The flow velocity was set to 0.35 mL per minute. The column oven was set to 40 °C. The injection volume was 4 μL. The effluent was alternatively connected to an electrospray ionization triple quadrupole linear ion trap mass spectrometer (ESI-Q-TRAP-MS).

#### 2.2.2. ESI-Q TRAP-MS/MS

LIT and triple quadrupole (QQQ) scans were acquired on a Q TRAP, AB4500 Q TRAP UPLC/MS/MS System, equipped with an ESI Turbo Ion-Spray interface, operating in positive- and negative-ion mode and controlled by Analyst 1.6.3 software (AB Sciex, Foster, CA, USA). The ESI source operation parameters were as follows: ion source, turbo spray; source temperature, 550 °C; ion spray voltage (IS), 5500 V (positive-ion mode)/−4500 V (negative-ion mode); ion source gas I (GSI), gas II(GSII), and curtain gas (CUR) set to 50, 60, and 25.0 psi, respectively; and high collision-activated dissociation (CAD). Instrument tuning and mass calibration were performed with 10 and 100 μmol/L polypropylene glycol solutions in the QQQ and LIT modes, respectively. The QQQ scans were acquired as MRM experiments with collision gas (nitrogen) set to medium. DP and CE for individual MRM transitions were performed with further DP and CE optimization. A specific set of MRM transitions were monitored for each period according to the metabolites eluted within this period [22].

### 2.3. Qualitative and Quantitative Result Analysis

From a scientific research perspective, this experimental workflow integrated the advantages of untargeted and targeted metabolomics. Specifically, qualitative analysis was performed using a high-resolution mass spectrometer (AB SCIEX TripleTOF 6600, SCIEX, Framingham, MA, USA) with plant-wide target detection, while quantitative analysis was conducted on the AB SCIEX 4500 QTRAP platform. The former enabled accurate metabolite identification through high-resolution mass spectrometry, whereas the latter complemented this with triple quadrupole technology, demonstrating high sensitivity, specificity, and superior quantitative capabilities.

Metabolite identification was established through four critical parameters: Q1 exact molecular mass (±20 ppm), MS/MS fragmentation patterns, retention time (RT ± 0.2 min), and isotopic distribution. The analytical process utilized both the MetWare database (MWDB) and the multiple-reaction monitoring (MRM) methodology. A proprietary intelligent MS/MS spectral-matching algorithm was employed to systematically compare experimental MS/MS spectra and retention times against database references, incorporating a mass error tolerance (MS/MS: 20 ppm) and a retention time deviation threshold (0.2 min).

For quantitative analysis, the QTRAP system operated in the MRM mode, monitoring five key parameters: de-clustering potential (DP), collision energy (CE), retention time (RT), precursor ion (Q1), and product ion (Q3). Data processing was performed using MultiQuant software (v3.0.3) to integrate chromatographic peaks and generate quantitative profiles of metabolites across biological samples. This dual-platform approach combined the exploratory power of high-resolution mass spectrometry with the precision of targeted quantitation, enabling comprehensive metabolic profiling while maintaining analytical rigor.

### 2.4. Statistical Analyses of Metabolite Data

All experiments were repeated three times, and the routine data were collated by Excel 2019, statistically analyzed using SPSS 24, and plotted and analyzed by the Hiplot platform. Principal component analysis (PCA) was carried out to compare samples with the R software version 4.3.2. Orthogonal partial least-squares discriminant analysis (OPLS-DA) was performed to detect differences in metabolites using ropls v1.19.8. In addition, permutation tests (200 replicates) were used to process the data using supervised models validated to avoid model overfitting. The differentially accumulating metabolites were identified with the |Log2FC| ≥  1 and VIP ≥  1 from the OPLS-DA model from different samples. In addition, a Student *t*-test was used (*p*  =  0.05) to assess the significance of these results.

All the metabolites were annotated by mapping to the KEGG database (http://www.kegg.jp/kegg/pathway.html, accessed on 30 October 2024), whereby the compound query corresponded to the KEGG data number C, the matched annotation pathway map number, and the enrichment analysis statistics for the number of metabolites in the pathway. The pathways were considered significantly enriched at *p*-values ≤  0.05.

## 3. Results

### 3.1. UPLC-MS/MS Analysis of Different Parts of Wax Gourds

#### 3.1.1. Quality Control, Statistics, and Metabolite Identification of Different Parts of Wax Gourd Samples

**Figure 1:**
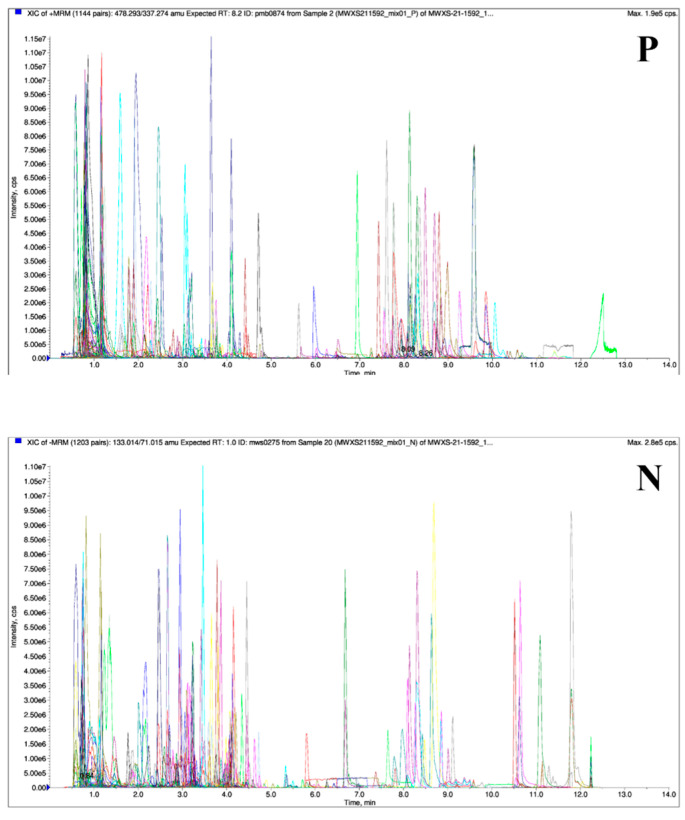
MRM metabolite assay multi-peak plot (P: positive-ion mode; N: negative-ion mode).

**Figure 2:**
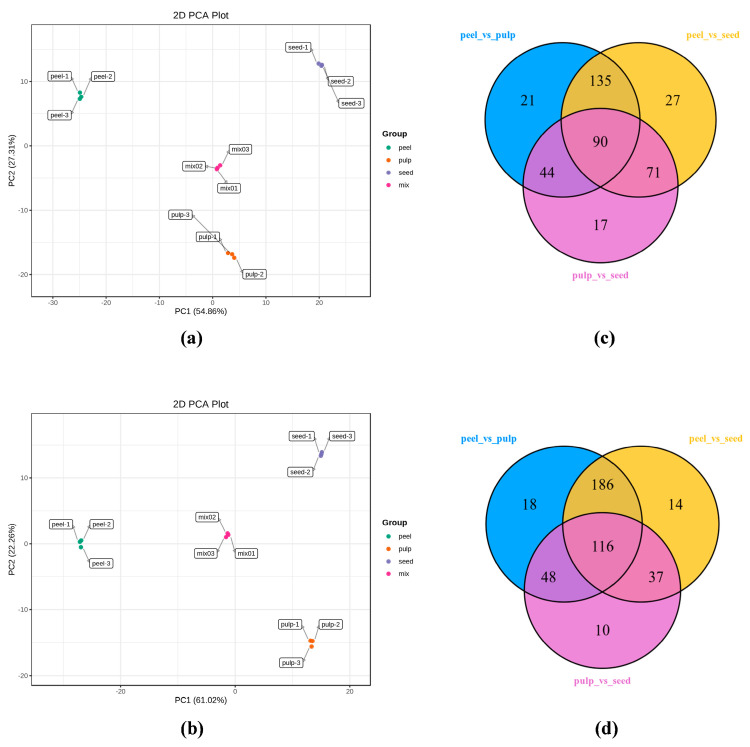
PCA and Venn diagram analyses of metabolites from different wax gourd parts (peel, pulp, seed, and mix, where the ratio of the three samples was 1:1:1): (**a**,**c**) primary differential metabolites’ PCA 2D result map and Venn diagram; and (**b**,**d**) secondary differential metabolites’ PCA 2D result map and Venn diagram.

As shown in Figure 1, the overlapping analysis of MRM metabolite detection multi-peak maps combined with the TIC diagram from the MS/MS of different quality-control (QC) samples showed that the ion peaks of the duplicate substances of the three samples were well overlapped, the MS/MS was stable, and the detection results were reliable. Through PCA of different samples, the results showed two principal components (PC1: 54.86%, PC2: 27.31%) of primary metabolites (Figure 2a) with a cumulative contribution of 82.17% and two principal components (PC1: 61.02%, PC2: 22.26%) of secondary metabolites (Figure 2b) with a cumulative contribution of 83.28%. Therefore, the PCA in this study could accurately reflect the main characteristics of the samples, and the PCA diagram shows the recognizable segregation trend of different samples, whereby the three groups vary significantly in metabolites. As shown in Table 1, 1020 metabolites were detected through qualitative and quantitative analyses of total metabolites in wax gourd peel, pulp, and seed, including 6 types of primary carbohydrates—amino acids and their derivatives, nucleotides and their derivatives, organic acids, lipids, carbohydrates and alcohols, and vitamins—accounting for 63.52% and 5 types of secondary metabolites, including flavonoids, phenolic acids, alkaloids, lignans, and coumarins, accounting for 36.48%. Then, 944, 905, and 934 metabolites were also identified, respectively, in the peel, pulp, and seed, among which the total abundance of metabolites was highest in the seeds, accounting for 39.14%, followed by the peel (32.94%) and the pulp (27.92%). The abundance of primary metabolites was rated as seed (26.62%) > peel (18.67%) > pulp (18.23%), while that secondary metabolites was peel (14.27%) > seed (12.52%) > pulp (9.69%). Among them, amino acids and their derivatives (18.33%), lipids (17.90%), and phenolic acids (19.86%) were the most abundant, while terpenoid metabolites (0.58%) were the least abundant.

#### 3.1.2. Screening for Differential Metabolites

Table 2 displays the findings related to the screening of differences in metabolites. There were 658 differential metabolites between the peel and the pulp: 290 of these were primary differential metabolites (133 up-regulated and 157 down-regulated), while 368 were secondary differential metabolites (133 up-regulated and 233 down-regulated). The 674 differential metabolites between the peel and seeds included 323 primary differential metabolites (207 up-regulated and 116 down-regulated) and 351 secondary differential metabolites (169 up-regulated and 182 down-regulated). Meanwhile, 433 differential metabolites were found between the pulp and seeds, with 224 primary differential metabolites (186 up-regulated and 38 down-regulated) and 209 secondary differential metabolites (162 up-regulated and 47 down-regulated). The main differential components between groups were amino acids and their derivatives, lipids, flavonoids, phenolic acids, and terpenoids, which were highly abundant and diverse.

#### 3.1.3. Analysis of Common Differential Metabolites

As shown in Figure 2c (the Venn diagram of common differential metabolites), there were 90 common differential metabolites among the primary metabolites of wax gourd peel, pulp, and seeds. Among them, 9(S)-hydroxy-10(E), 12Z-octadecadienoic acid, 17-hydroxy-linolenic acid, and 13-hydroxy-9,11-octadecadienoic acid were the most abundant in the peel, specifically 52.79–71.51 times and 14.44–30.33 times more abundant than in the pulp and seeds, respectively. In the pulp, 2-hydroxyisocaproic acid, alanine, and L-hype methionine were the highest, being 3.85~24.99 times and 3.72~10.30 times more abundant than in the peel and seeds, respectively. In the seed, citric acid, 2-hydroxy hexadecanoic acid, and lysophosphatidylcholine (15:0) were the most abundant, being 9.14~28.03 times and 2.90~8.78 times more abundant than in the peel and pulp, respectively.

As shown in Figure 2d, there were 116 common differential metabolites among the secondary metabolites of wax gourd peel, pulp and seeds. Among them, 3,4,5-trimethoxyphenyl-1-*O*-glucoside, phenylpropionic acid-*O*-β-d-glucopyranoside, and episyringaresinol 4′-*O*-β-d-glncopyranoside were the most abundant in the peel, with levels 11.52–105.57 times and 4.34–40.08 times higher than those in the pulp and seed, respectively. In the pulp, 2-hydroxy-3-phenylpropionic acid, 3-(4-hydroxyphenyl) propionic acid, and 2, 6-dimethoxybenzaldehyde were the highest, being 43.45~186.55 times and 10.87~14.15 times more abundant than in the peel and seeds, respectively. In the seeds, p-hydroxybenzoic acid, 2, 5-dihydroxybenzaldehyde, and salicylic acid were the most abundant, with levels 2.87~2.91 times and 7.25~7.86 times higher than those in the peel and pulp, respectively.

#### 3.1.4. Analysis of Differential Metabolites

**Figure 3:**
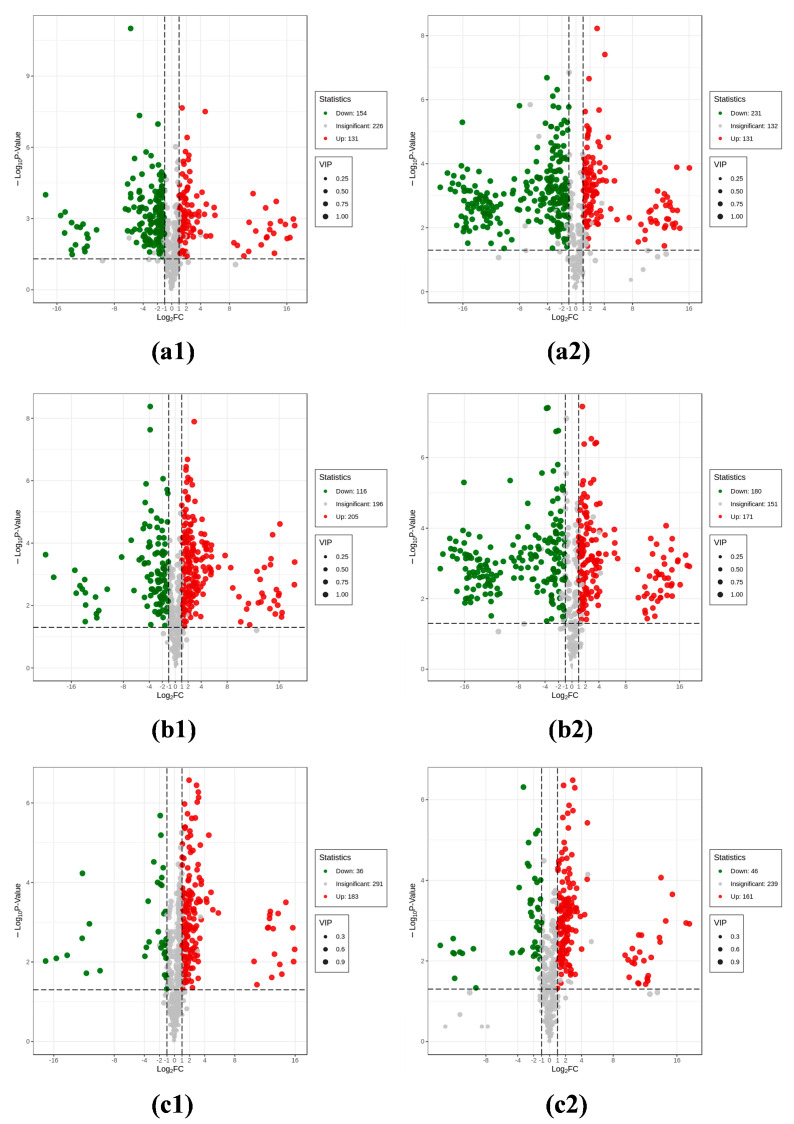
Analysis of differential metabolites in (**a**) peel vs. pulp, (**b**) peel vs. seed, and (**c**) pulp vs. seed, where (**a1**–**c1**) corresponds to primary differential metabolites and (**a2**–**c2**) to secondary differential metabolites.

Figure 3 depicts the metabolic screening results of the black wax gourd samples. Initially, among the primary metabolites, 285 distinct differences were observed between the peel and the pulp (154 up-regulated and 131 down-regulated), while 321 variations existed between the peel and the seeds (205 up-regulated and 116 down-regulated), and 219 distinctions were found between the pulp and the seeds (183 up-regulated and 36 down-regulated). Comparatively, among the secondary metabolites, there were 362 major differences between the peel and the pulp (131 up-regulated and 231 down-regulated), 351 between the peel and the seeds (171 up-regulated and 180 down-regulated), and 207 between the pulp and the seeds (161 up-regulated and 46 down-regulated). The primary differences included phenolic acids, amino acids and their derivatives, flavonoids, and lipids. The peel of black wax gourds, rich in phenolic acids and flavonoids, serves as a natural source of antioxidants. The pulp, with moderate levels of amino acids and lipids, is an excellent source of proteins and unsaturated fatty acids. Meanwhile, the seeds are vital raw materials for extracting bioactive components.

#### 3.1.5. Analysis of Unique Differential Metabolites in Peel vs. Pulp

As can be seen from Table 3, the unique differential metabolites for peel vs. pulp mainly included 21 primary differential metabolites: amino acid and their derivatives (5), nucleotides and their derivative (3), organic acid (5), lipid (3), carbohydrate, and alcohols (5). Among them, the content of leminoconic acid, 3-dehydro-l-threonic acid, l-malic acid, l-phenylalanine, 6-methylmercaptopurine, l-concanavaline sulfate, and (16: 3)-lysophosphatidylethanolamine was 2.09–3.21 times higher in the pulp of wax gourds (*p* < 0.05) compared to the peel. Meanwhile, the contents of l-threonine, adenine, adipic acid, 2-acetyl-2-hydroxybutyric acid, rhamnose, l-fucoitol, d-erythritose-4-phosphate, *N*-acetyl-l-phenylalanine, 5-l-glutamyl-l-amino acid, (7Z)-hexadecenoic acid, uridine 5′-monophosphate, d-fructose-1,6-diphosphate, lysophosphatidylethanolamine-16: 1 (2n isomeric), and adenyl succinic acid were significantly lower in the pulp of wax gourds (*p* < 0.05), corresponding to only one-quarter to one-half those in the peel. The unique differential metabolites of peel vs. pulp also included 18 different secondary metabolites: flavonoids (4), phenolic acids (8), alkaloids (3), lignans, and coumarin (3). Among them, the contents of *N*-phenylmethyleneisomethylamine, 1,3,5-pyrogallol, 7,8-dimethylpyrozine, isosaxifrin, isoimperatorin, feruloyl coumarin, gallyl methyl gallate, luteolin-7-*O*-neoorange peel glycoside, and chrysoeriol-7-*O*-rutinoside were significantly increased in the pulp of wax gourds (*p* < 0.05), being 2.20–10.44 times more abundant than in the peel. Meanwhile, agmatine, methyl p-coumarate, 3-hydroxy-1-(4-hydroxy-3,5-dimethoxyphenyl)propyl-1-ketone, 3,4′-dihydroxy-3′-methoxyphenylvaleric acid, protocatechuic acid-4-*O*-glucoside, 3,4-dihydroxyphenylethanol-β-d-glucoside, 3,5-dimethoxy-4-hydroxyphenol-1-*O*-glucoside, isoglycyrrhizin, and luteolin-7,3′-*O*-diglucoside were significantly decreased in the pulp of wax gourds (*p* < 0.05), amounting to less than one half of the content found in the peel.

#### 3.1.6. Analysis of Unique Differential Metabolites in Peel vs. Seeds

As can be seen from Table 4, the unique differential metabolites in peel vs. seeds mainly included 27 primary metabolites—amino acid and derivatives, lipid, carbohydrate and alcohols, and vitamins—among which cyclopentylglycine, allyl ester, isoxanthopterin, d-glucose, d-galactose, 4-pyridoxic acid, trimethyllysine, cyclic (proline-proline), l-tyrosine methyl ester, d-pantothenic acid, 9-hydroxy-12-oxo-15 (Z)-octadecenoic acid, rutose, pyridoxin-5′-*O*-glucoside, lactitol, tetraenylmenadione (vitamin K2), lysophosphatidylethanolamine 16: 0 (2n isomeric), lysophosphatidylethanolamine 16: 0, lysophosphatidylethanolamine 16: 0, lysophosphatidylethanolamine 18: 0, lysophosphatidylcholine 16: 2, lysophosphatidylcholine 16: 0 (2n isomeric), and lysophosphatidylcholine were significantly increased in the seeds of wax gourds (*p* < 0.05), being 2.02–5.71 times more abundant than in wax gourd peel. Meanwhile, the contents of d-threonate, sodium valproate, dodecanoic acid (lauric acid), 5-hydroxy-l-tryptophan, flavin mononucleotide (FMN), and 1-linoleoylglycerid-3-*O*-glucoside in wax gourd seeds were significantly decreased (*p* < 0.05), amounting to only one-sixth to one-half of those found in the peel. The contents of secondary unique differential metabolites, including 4-aminosalicylic acid, 4-hydroxybenzoate, bergamolide, 4-(3,4,5-trihydroxybenmethoxy) benzoic acid, bergapten, and 3′-demethylnobiletin, were significantly increased in the seeds of wax gourds (*p* < 0.05), being 2.04–4.18 times higher than those in the peel of wax gourds; meanwhile, the contents of genistein and corosoic acid in the seeds of wax gourd were significantly decreased (*p* < 0.05), amounting to less than 1/20 those in the peel. Methyl-5-glucosyloxy-2-hydroxybenzoate, 3-epiursolic acid, 3,5-di-*O*-caffeoylquinic acid, and 6′-*O*-sinapoylsucrose were unique to wax gourd peel, while 4-*O*-(6′-*O*-glucosyl-imino)-4-hydroxybenzyl alcohol and trans-Fertaric acid were unique to wax gourd seeds. In general, seeds had higher levels of primary metabolites like amino acids, lipids, carbohydrates, and vitamins, while some, like d-threonate and flavin mononucleotide, were lower. The contents of secondary metabolites like 4-aminosalicylic acid were also increased in the seeds, but the genistein content was decreased. Unique metabolites were found in both peel and seeds: methyl-5-glucosyloxy-2-hydroxybenzoate in the peel and 4-*O*-(6′-*O*-glucosyl-imino)-4-hydroxybenzyl alcohol in the seeds. These differences offer insights into wax gourds’ nutritional and bioactive properties.

#### 3.1.7. Analysis of Unique Differential Metabolites in Pulp vs. Seeds

As can be seen from Table 5, there were 17 compounds of primary unique differential metabolites in the pulp vs. seeds. Among them, the contents of 2-aminoisobutyric acid, *N*-acetyl-beta-alanine, isoguanine, xanthine, l-histidine, d-sorbitol, azelaic acid, l-homocystine, 9-hydroxy-10, 12-octadecadienoic acid ethyl ester, and d-(+)-cellobiose in wax gourd seeds were significantly increased (*p* < 0.05), being 2.02–7.56 times higher than in wax gourd pulp; meanwhile, the contents of cyclic (glycyl-l-alanyl), L-glutamine, N6-isopentene adenine, N-acetyl-l-arginine, ricinoleic acid, *N*-eicosanol, and maltotrisaccharide were significantly decreased in the seeds of wax gourds (*p* < 0.05), amounting to less than one-half those found in the pulp of wax gourds. In addition, there were 10 secondary unique differential metabolites in the pulp vs. seeds. Among them, the contents of choline, zeolactone, eucommidol, caffeoyltartaric acid, oleoylmonoethanolamine, feruloylsyringic acid, rosemarinic acid-3′-*O*-glucoside, and 3, 5-*O*-dicaffeoylquinic acid methyl ester were significantly increased in the seeds of wax gourds (*p* < 0.05), being 2.05–4.47 times higher than those in the pulp of wax gourds; meanwhile, the contents of 4′-hydroxy-3′-methoxyacetophenone and dihydroisocucurbitacin I-glucoside in wax gourd seeds were significantly decreased (*p* < 0.05), amounting to only one-quarter to two-fifths of those in the pulp of wax gourds. Seeds had higher levels of some primary metabolites like 2-aminoisobutyric acid and d-sorbitol, while others, like l-glutamine, were lower. Among the secondary metabolites, the levels of choline and zeolactone were increased in the seeds, but 4′-hydroxy-3′-methoxyacetophenone was decreased. These variations suggest unique nutritional and bioactive properties for each part of a wax gourd.

### 3.2. Differential Metabolite KEGG Enrichment Analysis

**Figure 4:**
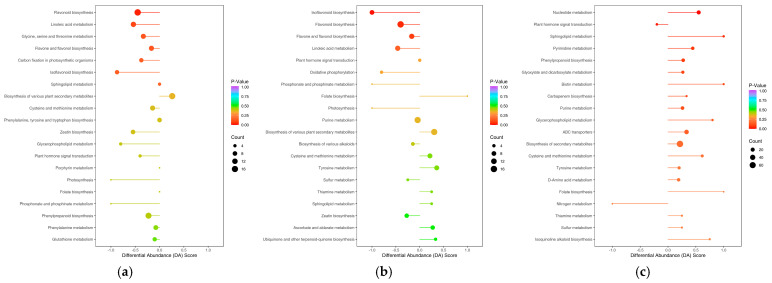
Enrichment map of differential metabolite KEGG: (**a**) peel vs. pulp; (**b**) peel vs. seed; and (**c**) pulp vs. seed.

The pathway analysis of differential metabolites in wax gourd tissues revealed distinct metabolic profiles between the peel and the pulp (Figure 4). The comparative analysis identified 85 metabolic pathways, with enrichment significance inversely correlated to p-value magnitude. The three most prominently enriched pathways were (1) flavonoid biosynthesis, (2) linoleic acid metabolism, and (3) glycine, serine, and threonine metabolism. A single pathway exhibited statistically significant differential enrichment (*p* < 0.05)—the flavonoid biosynthesis pathway. Within this pathway, 17 significant differential metabolites were noted, comprising 4 up-regulated and 13 down-regulated compounds. A tissue-specific analysis showed that the peel contained 16 metabolites participating in flavonoid biosynthesis, exceeding the pulp’s count by 3 metabolites. Notably, four metabolites displayed significant accumulation in the pulp: hesperidin-7-*O*-neohesperidin (hesperidin), hesperetin-7-*O*-glucoside, homoeriodictyol, and hesperetin (*p* < 0.05). Conversely, 13 metabolites showed significant depletion in the pulp compared to the peel, including chlorogenic acid (3-*O*-caffeoylquinic acid) and apigenin-8-C-glucoside (vitexin) (*p* < 0.05). The linoleic acid metabolism pathway contained 11 lipid-associated metabolites. Among these, linoleic acid and (9Z,11E)-octadecadienoic acid exhibited heightened metabolic activity and biosynthesis in pulp tissues. Ten differential metabolites were identified in glycine, serine, and threonine metabolism. Metabolic compartmentalization was observed: N-methylglycine and hydroxypyruvate showed no detectable synthesis in the pulp, while creatine was absent from the peel’s biosynthetic pathways. Tissue-specific accumulation patterns emerged, with L-aspartate and betaine preferentially accumulating in the pulp. Conversely, peel tissues demonstrated elevated levels of five metabolites: *N*,*N*-dimethylglycine, l-serine, l-homoserine, l-threonine, and 3-phospho-d-glycerate. These spatial metabolic distinctions suggest the differential regulation of amino acid metabolism between wax gourd peel and pulp tissues.

The differential metabolites in peel vs. seeds were found in 87 metabolic pathways, among which the top 3 pathways (Figure 4b) with the highest degree of enrichment (the closer the *p*-value was to 0, the more significant the enrichment) were those related to (1) isoflavone biosynthesis, (2) flavonoid biosynthesis, and (3) flavone and flavonol biosynthesis. Eight differentially significant metabolites were noted in the isoflavone biosynthesis pathway, in the significantly different metabolite pathway, while the differential seed metabolites in the isoflavone biosynthesis pathway were only genistein, naringin, and pratensein, with lower contents than those found in the peel. Apigenin, genistein, naringin, cherry flavin, chickpea A, pratensein, genistein, and 6′′-*O*-malonyl genistein all participated in the isoflavone biosynthesis of the peel. A total of 16 significantly different metabolites were linked to flavonoid biosynthesis in significantly different metabolic pathways (4 up-regulated and 12 down-regulated), and the synthesis of homoeriodictyol, hesperidin, hesperidin 7-*O*-glucoside, and hesperidin-7-*O*-neohesperidin (neohesperidin) was stronger in the seeds. A total of 10 metabolites were involved in the flavonoid and flavonol biosynthesis pathway: chrysoeriol, quercetin, apigenin-6-C-glucoside (isovitexin), apigenin-8-C-glucoside (vitexin), and vitexin-2′-*O*-rhamnoside were more synthesized in the peel; luteolin-7-*O*-glucoside (luteoloside) and quercein-3-*O*-rhamnoside (quercetin) were more strongly synthesized in the peel.

The comparative analysis of pulp versus seed tissues revealed differential metabolite distribution across 83 metabolic pathways (Figure 4c). Seed tissues exhibited enhanced activity in phenylalanine metabolism and carotenoid biosynthesis, while showing reduced activity in α-linoleic acid metabolism and plant growth regulation compared to the pulp. The phenylpropanoic acid biosynthesis pathway contained 16 distinct components, comprising 13 phenolic acids, 2 lignans/coumarins, and 1 alkaloid. The seed tissues demonstrated significantly elevated synthesis (2.27–6.78 fold) of multiple compounds, including 7-hydroxycoumarin, caffealdehyde, p-coumaric acid, p-caffeic aldehyde, caffeic acid, p-caffeitol, scopoletin lactone, ferulic acid, mustelin, 5-*O*-p-coumaroylquinic acid*, and chlorogenic acid (3-*O*-caffeoylquinic acid). Conversely, pulp tissues preferentially accumulated p-coumaral, cinnamic acid, p-coumaryl alcohol, and erucic acid. The sphingolipid metabolic pathway featured four differential components: l-serine (amino acid derivative), phosphoethanolamine (alkaloid), 3-dehydrosphingosine, and sphingosine 1-phosphate (lipids). All four metabolites showed significantly higher biosynthetic activity in the seeds relative to the pulp, indicating compartment-specific regulation of sphingolipid metabolism.

Therefore, amino acids and phenylpropanoids such as phenylalanine, tyrosine, glycine, serine, and threonine are mainly accumulated in wax gourd pulp; flavonoids are mainly accumulated in wax gourd peel; and nucleotide metabolism pathways are mainly accumulated in wax gourd peel and seeds.

## 4. Discussion

The metabolites of three parts (peel, pulp, and seeds) of wax gourds were determined and compared by ultra-performance liquid chromatography–tandem mass spectrometry (UPLC-MS/MS) with multivariate statistical analysis. A total of 944, 905, and 934 metabolites were screened, respectively, including primary metabolites (six major categories, such as amino acids and their derivatives, nucleotides and their derivatives, and organic acids) and secondary metabolites (five categories, such as flavonoids, phenolic acids, and alkaloids). The overall abundance of metabolites was observed to follow the order of seed > peel > pulp. Specifically, for the 404 primary metabolites, the abundance was highest in the seeds, followed by the peel and then the pulp. In contrast, for the 405 secondary metabolites, the abundance was highest in the peel, followed by the seeds and then the pulp. Notably, all three tissues (seeds, peel, and pulp) contained a relatively high number of metabolites classified as amino acids and their derivatives, lipids, and phenolic acids. The metabolites of different parts of the wax gourds differed significantly, with 658, 674, and 433 differential metabolites between the peel and the pulp, the peel and the seeds, and the pulp and the seeds, respectively. The primary differential components comprised amino acids and their derivatives, lipids, flavonoids, phenolic acids, and terpenes. Numerous compounds within these categories have been demonstrated to possess pharmacological activity and efficacy effects. For example, isoeucalyptus can reduce lipopolysaccharide-induced periodontitis by inhibiting the ERK1/2 and NF-κB pathways, and it has anti-inflammatory, analgesic, anti-tumor, anti-virus, and other pharmacological effects [23]; 3′-demethylated chuanchuanpirosin is a kind of polymethoxyflavonoid and a derivative of chuanhuangpirosin, which has been proven to be able to inhibit tumor angiogenesis and has an anti-tumor function [24]; meanwhile, the 3-*O*-glucoside of rosemary acid has a wide range of bioactivities, including antioxidant, antibacterial, anti-viral, antithrombotic, and immunosuppressive properties, etc. [25]. Genistein belongs to isoflavonoid components and can inhibit lipid accumulation in adipocytes by affecting the total cholesterol and triglyceride pathway, and it can also prevent the production of specific proteins in adipocytes, as well as the expression of genes responsible for the specific proteins [26]; p-hydroxybenzoic acid also exerts lipid-lowering effects by affecting the total cholesterol pathway [27]. However, the content of iso-eucalyptus in wax gourd pulp was 10.44 and 3.27 times higher than in the peel; the content of 3′-desmethylchondroitin in wax gourd seeds was 3.23 times higher than in the peel; and the content of the 3-*O*-glucoside of rosemarinic acid in wax gourd seeds was 4.47 times higher than in the pulp. Genistein was present in the peel and seeds of wax gourds, and p-hydroxybenzoic acid was present in the peel, pulp, and seeds of wax gourds, with the highest amount found in wax gourd seeds. Therefore, the differential metabolites between different parts of wax gourds contain many common active chemical components; however, whether the differences in efficacy are related to differences in the metabolites of the three parts hereby analyzed needs to be explored in further studies.

To gain a more specific understanding of the metabolites which contribute to the nutritional value of wax gourds, a total of 126 amino acids and their derivatives were detected in three parts of black wax gourds (peel, pulp, and seeds), with a relative content of 18.33%, including 20 basic amino acids and 8 essential amino acids.

Among them, the highest percentage was L-tryptophan (1.68%), which has a regulating effect on digestive function and protein synthesis [28,29], and a deficiency in this amino acid will cause reduced humoral immune function, diabetes mellitus, and neurological disorders [30]. Nucleotides and their derivatives are ubiquitous in all cells and play an important role in biological functions. They participate in cell growth and energy metabolism and play an important role in the body’s self-function and self-regulation [31]. The peel, pulp, and seeds of wax gourds contain a total of 69 nucleotides and their derivative metabolites, with a relative content of 9.55%, the highest contents being guanosine (2.79%) and adenine (1.77%). Guanosine plays a trophic and neuroprotective role in the organism. Studies have shown that it induces many beneficial cellular responses in several types of brain injuries, such as, for example, seizures, hypoxia, anxiety-like behaviors, ischemia, and glucose deprivation [32]. Adenine, one of the nucleobases used in the synthesis of nucleotides, plays a role in neuroprotection and injury perception, and its deficiency leads to intellectual disability, blood disorders, and kidney disorders, among others [33]. Wax gourd peel, pulp, and seeds were also screened for 142 lipids, amounting to 17.90% of free fatty acids, glycerides, lysophosphatidylcholine, etc., the most abundant being linoleic acid (1.32%), a functional polyunsaturated fatty acid which is essential in the body. The vitamins (2.06%) in the peel, pulp, and seeds of wax gourds were mainly vitamins B and K. Niacinamide has anti-inflammatory properties and is involved in processes related to cell cycle regulation [34], DNA repair, and lipid metabolism, among others [35]. Vitamin B6, which was found to have a high content in our study (0.15%), is involved in the metabolism of amino acids and the synthesis of hemoglobin [19]. The sugar content of wax gourds was 5.24%, composed of D-fructose, D-glucose, D-mannose, D-galactose, and so on. Organic acids were widely distributed in all parts of wax gourds, with a content of up to 9.89%, including 89 types, such as succinic acid, citric acid, propanedioic acid, 2,3-dihydroxy-3-methylbutanoic acid, and piperidinic acid, which play an important role in intrinsic qualities such as taste and flavor [36].

The metabolites that contribute to the functional activity value of wax gourds are malonyl glycolic acid, inositol, levulinic acid, etc. Malonyl glycolic acid is a small-molecule organic acid (1.10%) with a high relative content in wax gourds, which can effectively inhibit the conversion of sugar into fat and reduce fat accumulation. In addition, it is also one of the important indices used to measure the health function of raw materials and their processed products [37]. γ-aminobutyric acid, with a content of 0.22% in the wax gourd samples in our study, is an important inhibitory neurotransmitter, which is effective in mental disorders, respiratory diseases, cardio–cerebral and cerebral–vascular diseases, etc., as well as a variety of physiological functions, such as immunity and hormone regulation [38]. With a 0.05% content in the wax gourd samples in our study, inositol is vital to most biological systems. It can not only lower blood lipids, promote cholesterol metabolism, and have antioxidant, anticancer, and antidiabetic functional activities, but it can also be used as a nutrient in the food industry. L-carnitine and fenugreek alkaloids are alkaloids with a high content in wax gourds [39] that play an important role in the physiological function of the organism. Some studies have demonstrated that L-carnitine is just a vitamin-like substance that has various physiological functions, such as fat oxidation and decomposition, weight loss, anti-fatigue effects, etc. [40]. It can be used as a food additive in weight-loss food, athletes’ food, etc. The content of this substance in wax gourds is as high as 2.08%, so wax gourds can lower the fat content and reduce one’s weight. Therefore, the main active substances in wax gourds with lipid-lowering efficacy are malonyl glycolic acid and L-carnitine. The content of fenugreek alkaloids in wax gourds is 1.02%, and existing studies have shown that these substances have the effect of regulating blood glucose and blood lipids, as well as anticancer properties and so on [41].

In summary, this study was the first to use widely targeted metabolomic technology to isolate and identify the metabolites in wax gourds, analyze their chemical composition, primary metabolites (nutrients), and secondary metabolites (active ingredients), and explore the value of key differential metabolites in different parts of these plants, so as to provide a theoretical basis for the comprehensive processing and utilization of black wax gourds. However, wax gourds contain many active metabolites that can be extracted and utilized, with differences in metabolites between different plant parts which need to be further explored to discover their potential value.

## 5. Conclusions

The abundance of metabolites in each part of the wax gourds was significantly different, with the metabolite content in the seeds being much higher than in the peel and the pulp. The most abundant metabolite classes in wax gourd peel and seeds were phenolic acids and lipids, respectively, and the most abundant metabolite class in the pulp was that of amino acids and their derivatives. Among the 11 kinds of metabolites found in wax gourds, the following were the most abundant in the seeds: lipids, alkaloids, amino acids and their derivatives, organic acids, sugars and alcohols, and vitamins. Meanwhile, the most abundant metabolites in the peel were flavonoids, phenolic acids, terpenoids, lignans, and coumarins. High levels of L-tryptophan, guanosine, linoleic acid, malonic acid, γ-aminobutyric acid, inositol, levulinic acid, fenugreek alkaloids, and other metabolites were found in wax gourds, contributing significantly to their nutritional value and functional activity. The results of this study can provide a reference for the thorough processing and quality regulation of wax gourds, which are of great significance for promoting the high-quality development of the wax gourd industry.

## Figures and Tables

**Table 1:** Metabolites of different parts of wax gourds are summarized.

No.		Metabolite Category	Quantity (Relative Content %)	Number of Metabolites (Relative Content %)		
				Peel	Pulp	Seed
Primary metabolites	1	Amino acids and derivatives	126 (18.33 ± 0.12)	117 (5.32 ± 0.08)	121 (6.14 ± 0.03)	124 (6.87 ± 0.03)
	2	Nucleotides and derivatives	69 (9.55 ± 0.15)	65 (3.91 ± 0.08)	68 (1.58 ± 0.01)	69 (4.06 ± 0.22)
	3	Organic acids	89 (9.89 ± 0.15)	81 (2.80 ± 0.04)	86 (3.17 ± 0.14)	87 (3.92 ± 0.07)
	4	Lipids	142 (17.90 ± 0.10)	141 (4.79 ± 0.07)	134 (4.84 ± 0.10)	136 (8.27 ± 0.12)
	5	Sugars and alcohols	73 (5.79 ± 0.07)	65 (1.32 ± 0.02)	67 (1.88 ± 0.03)	69 (2.59 ± 0.10)
	6	Vitamins	21 (2.06 ± 0.06)	21 (0.53 ± 0.00)	19 (0.62 ± 0.05)	20 (0.91 ± 0.02)
		Total	520 (63.52 ± 0.43)	490 (18.67 ± 0.10)	493 (18.23 ± 0.20)	505 (26.62 ± 0.27)
Secondary metabolites	7	Flavonoids	149 (7.29 ± 0.15)	136 (3.38 ± 0.05)	114 (1.86 ± 0.05)	117 (2.05 ± 0.06)
	8	Phenolic acids	204 (19.86 ± 0.27)	187 (7.59 ± 0.05)	171 (5.19 ± 0.17)	182 (7.08 ± 0.16)
	9	Alkaloids	61 (7.00 ± 0.02)	57 (2.01 ± 0.01)	58 (2.26 ± 0.04)	60 (2.73 ± 0.05)
	10	Lignans and coumarins	61 (1.75 ± 0.04)	53 (0.95 ± 0.05)	54 (0.26 ± 0.01)	55 (0.54 ± 0.01)
	11	Terpenoids	25 (0.58 ± 0.04)	21 (0.34 ± 0.01)	13 (0.12 ± 0.04)	15 (0.12 ± 0.01)
		Total	500 (36.48 ± 0.48)	454 (14.27 ± 0.10)	410 (9.69 ± 0.25)	429 (12.52 ± 0.25)
		metabolites	1020 (100)	944 (32.94 ± 0.00)	905 (27.92 ± 0.06)	934 (39.14 ± 0.06)

**Table 2:** Statistical table of different metabolites between groups of wax gourd parts.

	No.	Category	Peel vs. Pulp	Peel vs. Seed	Pulp vs. Seed
			Total (Up/Down)	Total (Up/Down)	Total (Up/Down)
Primary metabolites	1	Amino acids and their derivatives	62 (38/24)	77 (61/16)	60 (47/13)
	2	Nucleotides and their derivatives	41 (5/36)	40 (18/22)	41 (36/5)
	3	Organic acids	56 (30/26)	55 (36/19)	37 (29/8)
	4	Lipids	88 (41/47)	103 (60/43)	60 (54/6)
	5	Sugars and alcohols	36 (17/19)	35 (23/12)	20 (15/5)
	6	Vitamins	7 (2/ 5)	13 (9/4)	6 (5/1)
	Total		290 (133/157)	323 (207/116)	224 (186/38)
Secondary metabolites	1	Flavonoids	123 (45/78)	121 (49/72)	33 (25/8)
	2	Phenolic acids	148 (51/97)	135 (73/62)	103 (77/26)
	3	Alkaloids	32 (13/19)	27 (16/11)	28 (24/4)
	4	Lignans and coumarins	47 (22/25)	46 (25/21)	34 (27/7)
	5	Terpenoids	18 (4/14)	22 (6/16)	11 (9/2)
	Total		368 (135/233)	351 (169/182)	209 (162/47)
	Total differential metabolites		658 (268/390)	674 (376/298)	433 (348/85)

**Table 3:** Peel- vs. pulp-specific differences in metabolites.

ID	*m/z*		Name of Fold Change (Pulp/Peel)	Chromatographic Peak Areas		VIP	Difference Multiple	*p*-Value	Type
	Q1	Q3		**Peel**	**Pulp**				
Primary Differential Metabolites									
mws0230	120.07	74	l-Threonine	4,503,833 ± 152,026	1,546,467 ± 41,357	1.1	0.34	0	Down
mws0425	129.02	85	Citraconic acid	86,139 ± 16,658	221,163 ± 21,215	1.06	2.57	0	Up
Lmbn000198	133.01	71.01	3-Dehydro-l-threonic acid	413,356 ± 16,314	902,200 ± 137,376	1.07	2.18	0.02	Up
mws0275	133.01	71.01	l-malic acid	409,073 ± 30,929	853,487 ± 81,403	1.08	2.09	0.01	Up
pme0040	136.06	119	Adenine	69,214,000 ± 2,743,185	29,226,333 ± 1,229,554	1.09	0.42	0	Down
mws0208	145.05	101	Adipic acid	1,581,100 ± 42,670	730,963 ± 42,042	1.09	0.46	0	Down
Lmbn001609	145.05	83.05	2-Acetyl-2-hydroxybutyric acid	281,627 ± 32,358	139,250 ± 7700	1.07	0.49	0.01	Down
mws0854	163	59.01	Rhamnose	213,700 ± 42,067	61,154 ± 7047	1.07	0.29	0.02	Down
pme0021	166.09	120.08	l-Phenylalanine	899,753 ± 16,929	1,879,000 ± 35,172	1.1	2.09	0	Up
pmc0274	167.1	121.1	6-Methylmercaptopurine	3,258,100 ± 76,300	7,710,100 ± 278,967	1.1	2.37	0	Up
MWSmce165	165.08	59.02	l-Fucose	1,901,633 ± 83,155	738,243 ± 101,290	1.08	0.39	0	Down
Zmzn000079	199	78.96	d-Erythrose-4-phosphate	103,930 ± 16,989	28,742 ± 3662	1.08	0.28	0.01	Down
Zmgn002106	206.08	58.03	*N*-Acetyl-l-phenylalanine	371,247 ± 69,554	165,817 ± 4643	1.05	0.45	0.04	Down
pme2566	217.08	199	5-l-glutamyl-l-amino acid	54,064 ± 3577	22,579 ± 5356	1.05	0.42	0	Down
Lmcn009122	253.22	235.21	(7Z)-hexadec-10-enoic acid	32,369 ± 3598	15,783 ± 1431	1.07	0.49	0.01	Down
MWSmce356	275.07	114.02	l-tryptophan sulfate	48,404 ± 6298	155,297 ± 13,533	1.08	3.21	0	Up
pme3188	323.03	211	Uridine 5′-monophosphate	8,032,433 ± 276,343	2,456,367 ± 243,901	1.09	0.31	0	Down
pme3311	339	97	d-fructose-1,6-diphosphate	92,208 ± 3048	25,099 ± 7497	1.05	0.27	0	Down
Lmhp007836	448.25	307.23	Lysophosphatidylethanolamine 16:3	5298 ± 1586	11,567 ± 1600	1	2.18	0.01	Up
Lmhp008763	452.28	311.26	Lysophosphatidylethanolamine 16:1 (2n isomer)	3,094,700 ± 135,834	1,236,767 ± 131,562	1.09	0.4	0	Down
MWS0155	462.07	96.97	Adenosine-5′-monophosphate	57,563 ± 3035	27,998 ± 5880	1.03	0.49	0	Down
Secondary Differential Metabolites									
pmp001287	120.08	103.05	N-benzylidene methylamine	9,232,867 ± 243,854	20,316,333 ± 498,097	1.06	2.2	0	Up
NK10264324	127.04	81.03	1,3,5-Benzenetriol	172,853 ± 22,483	396,137 ± 43,430	1.03	2.29	0	Up
pmb0501	131.13	114.1	Butylguanidine	84,127 ± 9112	29,414 ± 3450	1.04	0.35	0	Down
mws1195	179.07	147.1	Methyl p-coumarate	316,540 ± 4518	156,163 ± 3780	1.06	0.49	0	Down
Lmyp003951	227.09	181.09	3-Hydroxy-1-(4-hydroxy-3,5-dimethoxyphenyl)propan-1-one	728,393 ± 62,642	339,023 ± 20,476	1.04	0.47	0	Down
Lmzn001925	153.02	109.03	3,4′-Dihydroxy-3′-methoxybenzenepentanoic acid	32,558 ± 7055	13,457 ± 1146	1.01	0.41	0.04	Down
mws1383	243.09	145	7,8-Dimethylxanthine	51,294 ± 5875	124,537 ± 5641	1.04	2.43	0	Up
MWSslk172	247.06	217.02	Isorhoifolin	2696 ± 1425	28,141 ± 2149	1.01	10.44	0	Up
pmf0526	271.1	147.2	Isorhoifolin	2398 ± 595	7845 ± 1202	1.02	3.27	0.01	Up
pmn001367	315.07	153.02	Protocatechuic acid 4-*O*-glucoside	12,788,000 ± 25,229	5,425,533 ± 280,837	1.05	0.42	0	Down
Jmwn002172	331.1	153.02	3,4-Dihydroxyphenylethanol-β-d-glucopyranoside	269,997 ± 20,971	24,212 ± 8396	1.04	0.09	0	Down
pmb0235	323.09	177.4	Feruloylcoumarin	40,454 ± 5226	95,878 ± 8366	1.03	2.37	0	Up
Lmmn001294	327.14	165.09	3,5-Dimethoxy-4-hydroxyphenol-1-*O*-glucoside	3,362,700 ± 153,119	964,387 ± 101,376	1.05	0.29	0	Down
pmn001519	183.03	124.02	Methyl gallate	31,494 ± 5903	94,635 ± 3909	1.04	3	0	Up
pmp000384	419.13	257.08	Iso-Glycitin	41,081 ± 6680	8902 ± 2384	1.03	0.22	0.01	Down
pmp001079	609.18	301	Luteolin-7-*O*-neohesperidoside	32,514 ± 4815	78,608 ± 5825	1.03	2.42	0	Up
pmb3002	595.17	449.1	Luteolin-7-*O*-rutinoside	116,570 ± 12,194	373,607 ± 11,425	1.05	3.2	0	Up
Zmxp003107	458.1	116.3	Luteolin-7,3′-*O*-diglucoside	73,390 ± 13,231	28,595 ± 3214	1.02	0.39	0.02	Down

**Table 4:** Peel- vs. seed-specific differences in metabolites.

ID	*m*/*z*		Name of Fold Change (Peel/Seed)	Chromatographic Peak Areas		VIP	Difference Multiple	***p*-Value**	**Type**
	Q1	Q3		Peel	Seed				
Primary Differential Metabolites									
mws0889	135.03	75.01	d-threonic acid	3,411,633 ± 221,075	1,192,733 ± 103,148	1.07	0.35	0	Down
Hmsp000364	144.1	98.09	Cyclopentylglycine	171,100 ± 20,154	353,580 ± 19,226	1.05	2.07	0	Up
pme3351	144.1	126	Allyl ester	42,915 ± 9746	126,007 ± 16,787	1.04	2.94	0	Up
mws0851	143.11	143.11	Sodium valproate	1,235,133 ± 38,875	59,2097 ± 13,556	1.07	0.48	0	Down
mws0981	178.04	136.02	Isoxanthopterin	30,873 ± 5210	74,217 ± 4327	1.05	2.4	0	Up
mws4170	179.06	59.01	d-glucose	9,070,467 ± 1,228,309	29,221,000 ± 1,467,887	1.07	3.22	0	Up
pmf0139	179.06	59	d-galactose	6,565,200 ± 361,045	20,125,667 ± 666,863	1.07	3.07	0	Up
pme2596	170.08	134.1	4-pyridoxine	233,487 ± 7043	552,763 ± 3316	1.08	2.37	0	Up
Zmzp000145	189.16	84.08	Trimethyllysine	1,996,867 ± 378,707	5,128,667 ± 224,260	1.05	2.57	0	Up
Lmrj002244	195.11	70.06	Cyclo(proline-proline)	10,155 ± 1392	27,690 ± 7078	1.02	2.73	0.05	Up
MWS1771	196.1	91.05	l-tyrosine methyl ester	145,483 ± 14,822	404,253 ± 17,053	1.07	2.78	0	Up
pmb2640	199.17	199.17	Lauric acid	286,630 ± 15,541	125,020 ± 7052	1.07	0.44	0	Down
mws1337	220.12	202.1	d-pantothenic acid	9,018,600 ± 310,680	18,228,000 ± 364,037	1.07	2.02	0	Up
pme1228	221.09	204	5-hydroxy-l-tryptophan	167,030 ± 20,955	69,374 ± 4561	1.06	0.42	0.01	Down
pmn001689	293.21	235.17	9-hydroxy-12-oxo-15(Z)-octadecenoic acid	9273 ± 169	22,291 ± 2785	1.06	2.4	0.01	Up
Lmqn000351	325.11	59.01	Rhamnose	48,141 ± 11,530	111,822 ± 16,984	1.01	2.32	0.01	Up
pmb0789	332.13	152.07	Pyridoxal-5′-*O*-glucoside	494,887 ± 95,367	1,263,000 ± 100,484	1.04	2.55	0	Up
MWSslk074	343.12	59.01	Lactitol	4,802,767 ± 98,615	10,730,000 ± 226,316	1.08	2.23	0	Up
pme1014	445.3	341.3	Menatetrenone (vitamin K2)	112,310 ± 3104	238,977 ± 14,500	1.07	2.13	0	Up
pmd0160	496.34	184.07	Lysophosphatidylethanolamine 16:0 (2n isomer)	333,030 ± 18,370	890,643 ± 25,151	1.07	2.67	0	Up
pmb0876	483.27	255.23	Lysophosphatidylethanolamine 16:0	337,597 ± 30,415	878,040 ± 30,869	1.07	2.6	0	Up
MWS5083	455.1	96.97	Fumarate mononucleotide (FMN)	237,953 ± 20,041	93,554 ± 26,151	1.01	0.39	0	Down
pmb0883	482.32	341.31	Lysophosphatidylethanolamine 18:0	408,780 ± 41,361	1,060,963 ± 218,639	1.04	2.6	0.03	Up
pma1303	492.31	184.07	Lysophosphatidylcholine 16:2	412,553 ± 7690	1,303,500 ± 12,217	1.08	3.16	0	Up
pmb0863	454.29	313.27	Lysophosphatidylcholine 16:0 (2n isomers)	11,407,000 ± 304,242	36,728,000 ± 416,476	1.08	3.22	0	Up
Lmhp010573	517.34	263.24	1-linoleoylglycerol-3-*O*-glucoside	94,823 ± 13,666	16,682 ± 2030	1.07	0.18	0.01	Down
pmp001281	522.36	184.07	Lysophosphatidylcholine 18:1	9,784,400 ± 513,013	38,107,000 ± 637,846	1.07	5.71	0.01	Up
Secondary Differential Metabolites									
ML10177402	152.03	108.05	4-aminosalicylic acid	20,380 ± 5761	63,312 ± 8733	1	3.11	0	Up
MWS1839	165.06	92.03	Ethyl 4-hydroxybenzoate	16,106 ± 1936	51,140 ± 4049	1.04	3.18	0	Up
mws1071	217.05	201.4	Bergamottin	16,066 ± 2629	32,993 ± 2524	1.01	2.05	0	Up
mws0063	271.06	153.02	Genistein	291,050 ± 3581	13,189 ± 1626	1.05	0.05	0	Down
Lmyn003835	289.04	137.02	4-(3,4,5-trihydroxybenzyloxy)benzoic acid	12,071 ± 2540	504,17 ± 9587	1.03	4.18	0.02	Up
Lmmn003663	329.09	167.04	Methyl 5-glucoxy-2-hydroxybenzoate	158,563 ± 27,550	ND	1.05	0	0.01	Down
Jmbp006554	389.12	359.07	Ethyl rosmarinate	102,944 ± 5198	210,320 ± 7153	1.05	2.04	0	Up
Lmcp007265	389.12	359.08	3′-demethylnobiletin	35,375 ± 587	114,287 ± 3024	1.05	3.23	0	Up
MWSmce052	455.36	455.36	3-epibenzoic acid	456,187 ± 40,667	ND	1.05	0	0	Down
Zmhn003106	461.15	337.09	4-*O*-(6′-*O*-glucopyranosyl-imino)-4-hydroxybenzyl alcohol	ND	248,107 ± 28,303	1.05	27,567.41	0	Up
Zmpn008194	471.35	471.35	Corosolic acid	1,465,757 ± 596,114	10,446 ± 2121	1.05	0.01	0.05	Down
Lmhn003799	501.1	307.05	Feruloylferulic acid feruloyl-l-tartaric acid	ND	55,070 ± 3954	1.05	6118	0	Up
Wmzn002116	515.12	353.09	3,5-dicaffeoylquinic acid	125,287 ± 5973	ND	1.05	0	0	Down
Lmsn003628	547.17	223.1	6′-*O*-sinapoylsucrose	289,620 ± 47,716	ND	1.05	0	0.01	Down

ND: Represents the not detected in samples.

**Table 5:** Pulp- vs. seed-specific differences in metabolites.

ID	*m*/*z*		Name of Fold Change (Pulp/Seed)	Chromatographic Peak Areas		VIP	Difference Multiple	*p*-Value	Type
	Q1	Q3		Pulp	Seed				
Primary Differential Metabolites									
pme3017	104.07	58.00	2-aminoisobutyric acid	2,619,800 ± 204,823	7,807,867 ± 772,368	1.12	2.98	0	Up
MWSmce706	129.07	56.05	Cyclo(glycyl-l-alanyl)	262,900 ± 32,361	102,312 ± 13,370	1.1	0.39	0.01	Down
MWS2413	130.05	58.03	*N*-acetyl-β-alanine	25,521 ± 3942	70,898 ± 15,610	1.08	2.78	0.03	Up
pme0193	147.08	84.00	L-glutamine	170,230 ± 239,687	52,551 ± 17,783	1.07	0.31	0	Down
pme0183	152.06	135.00	Isoguanine	63,000 ± 5290	163,067 ± 21,330	1.11	2.59	0.01	Up
pme0256	151.03	151.00	Xanthine	232,217 ± 58,144	704,923 ± 174,233	1.06	3.04	0.03	Up
mws0254	156.08	110.00	l-histidine	5,254,033 ± 276,779	10,624,967 ± 769,176	1.11	2.02	0	Up
mws0214	181.07	71.00	d-sorbitol	97,320 ± 5823	201,470 ± 2260	1.12	2.07	0	Up
mws0237	187.10	125.00	Azelaic acid	6,140,633 ± 240,694	12,480,667 ± 456,462	1.12	2.03	0	Up
pme2060	204.25	136.00	N6-isopentenyladenine	38,353 ± 7567	17,936 ± 4052	1.01	0.47	0.02	Down
pme0170	217.13	158.00	*N*-acetyl-l-arginine	116,436 ± 18,342	57,584 ± 6193	1.07	0.49	0.02	Down
pme2890	269.06	136.00	l-cysteine	5680 ± 1368	42,967 ± 3715	1.11	7.56	0	Up
Lmbn009444	297.24	183.14	Ricinoleic acid	81,700 ± 5000	37,971 ± 1328	1.12	0.46	0	Down
pmf0297	297.32	183.10	n-docosanol	78,398 ± 6882	35,779 ± 730	1.12	0.46	0.01	Down
Hmqp006023	326.27	309.27	Ethyl 9-hydroxy-10,12-octadecadienoate	17,511 ± 3640	46,363 ± 1003	1.09	2.65	0	Up
MWSmce203	341.12	73.03	d-(+)-cellobiose	19,646 ± 3737	50,640 ± 14,435	1.04	2.58	0.06	Up
MWS0442	527.16	365.10	Maltotriose	130,434 ± 35,925	56,861 ± 5581	1.03	0.44	0.07	Down
Secondary Differential Metabolites									
pmb0484	104.10	60.10	Choline	519,1300 ± 20,2733	15,198,333 ± 362,467	1.14	2.93	0	Up
mws1382	165.06	122.04	4′-hydroxy-3′-methoxyacetophenone	91,188 ± 10,580	33,927 ± 4393	1.12	0.37	0.01	Down
pmn001492	187.10	123.10	Zelandine	394,527 ± 27,333	824,017 ± 19,184	1.13	2.09	0	Up
pmn001380	187.10	169.09	Eucommiol	630,580 ± 20,976	1,292,967 ± 50,931	1.14	2.05	0	Up
Lmhn001477	311.04	179.04	Caffeoyl tartaric acid	17,671 ± 2004	40,448 ± 2249	1.12	2.29	0	Up
mws0983	326.31	62.06	Oleoyl monoethanolamine	135,163 ± 30,152	340,493 ± 7545	1.09	2.52	0	Up
pmb0108	375.20	137.10	Feruloyl syringic acid	73,136 ± 10,371	1566,00 ± 21,570	1.09	2.14	0.01	Up
pmn001710	521.13	359.08	Rosmarinic acid 3′-*O*-glucoside	169,020 ± 35,578	755,923 ± 108,774	1.12	4.47	0.01	Up
Lmjp003822	531.15	177.06	3,5-*O*-bis-caffeoylquinic acid methyl ester	32,926 ± 3284	115,763 ± 8785	1.13	3.52	0	Up
mab0299	677.35	659.35	Dihydroisocoumarin I-glucoside	166,360 ± 21194	48,042 ± 6209	1.12	0.29	0.01	Down

## Data Availability

The original contributions presented in this study are included in the article/Supplementary Materials; further inquiries can be directed to the corresponding authors.

## Supplementary Materials

The following supporting information can be downloaded at https://www.mdpi.com/article/10.3390/foods14061046/s1: File S1, including Table S1, featuring basic indicators of black-skinned wax gourd, and Figure S1, showing the OPLS-DA model validation plot; and File S2, comprising all sample data.
