# Supplementary material for: Metabolomic Analysis of Different Parts of Black Wax Gourd (Cucurbita pepo)
Source: Foods. 2025 Mar 19;14(6):1046. doi: 10.3390/foods14061046 (PMC11941785; doi:10.3390/foods14061046)
Supplement: Supplementary file 1 [file foods-14-01046-s001.zip › File S1. Supplementary data.pdf]

Table S1 Basic indicators of black-skinned wax gourd

	Black-skinned winter melon	
Appearance Index	Vertical diameter（cm）	87.33±2.52
	Transverse diameter（cm）	19.00±1.00
	Thickness of pulp(cm)	6.63±1.09
	Weight per fruit(kg)	14.44±0.66
	Fruit shape index	4.60±0.20
	Fruit firmness(kg/cm)	2.51±0.15
Fruit composition	Skin content	10.05±1.15
	Seed content	0.55±0.08
	Edibility	87.08±0.53
	Juice yield	54.99±1.68
	Flesh moisture content	95.71±0.36
	Winter Melon Powder	4.74±0.45
pH		5.50±0.03
Soluble solids/(°Brix)		2.83±0.01
Total juice acid/(g/kg)		1.54±0.04
Cooler	L	16.32±0.30
	a	-0.71±0.12
	b	-0.03±0.05

Figure S1 OPLS-DA model validation plot

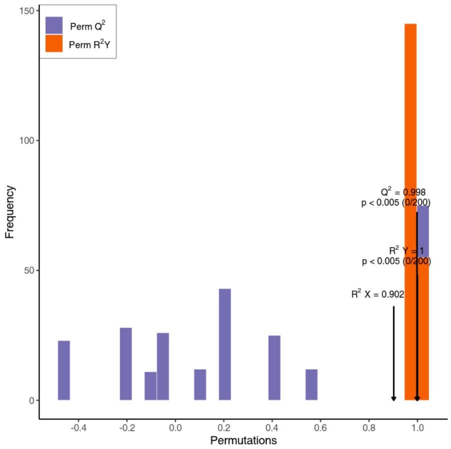

t

(1) peel_vs_pulp_opls_permutation

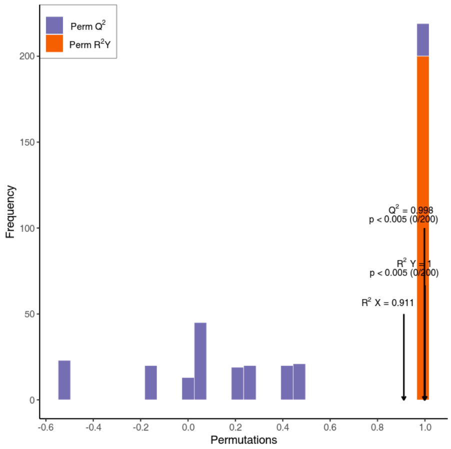

(2) peel_vs_seed_opls_permutation

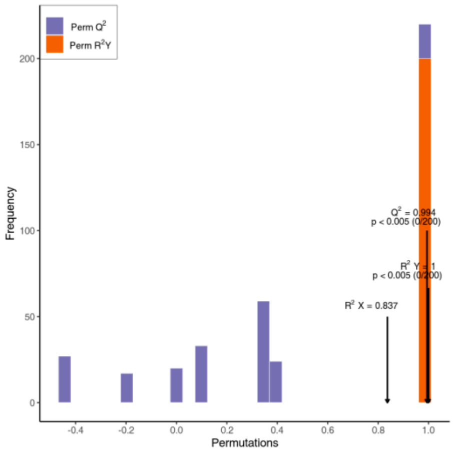

(3) pulp_vs_seed_opls_permutation
